# Assessment of instantaneous cognitive load imposed by educational multimedia using electroencephalography signals

**DOI:** 10.3389/fnins.2022.744737

**Published:** 2022-08-01

**Authors:** Reza Sarailoo, Kayhan Latifzadeh, S. Hamid Amiri, Alireza Bosaghzadeh, Reza Ebrahimpour

**Affiliations:** ^1^Artificial Intelligence Group, Faculty of Computer Engineering, Shahid Rajaee Teacher Training University, Tehran, Iran; ^2^School of Cognitive Sciences, Institute for Research in Fundamental Sciences, Tehran, Iran

**Keywords:** instantaneous cognitive load, EEG, classification, educational video, multimedia design

## Abstract

The use of multimedia learning is increasing in modern education. On the other hand, it is crucial to design multimedia contents that impose an optimal amount of cognitive load, which leads to efficient learning. Objective assessment of instantaneous cognitive load plays a critical role in educational design quality evaluation. Electroencephalography (EEG) has been considered a potential candidate for cognitive load assessment among neurophysiological methods. In this study, we experiment to collect EEG signals during a multimedia learning task and then build a model for instantaneous cognitive load measurement. In the experiment, we designed four educational multimedia in two categories to impose different levels of cognitive load by intentionally applying/violating Mayer’s multimedia design principles. Thirty university students with homogenous English language proficiency participated in our experiment. We divided them randomly into two groups, and each watched a version of the multimedia followed by a recall test task and filling out a NASA-TLX questionnaire. EEG signals are collected during these tasks. To construct the load assessment model, at first, power spectral density (PSD) based features are extracted from EEG signals. Using the minimum redundancy - maximum relevance (MRMR) feature selection approach, the best features are selected. In this way, the selected features consist of only about 12% of the total number of features. In the next step, we propose a scoring model using a support vector machine (SVM) for instantaneous cognitive load assessment in 3s segments of multimedia. Our experiments indicate that the selected feature set can classify the instantaneous cognitive load with an accuracy of 84.5 ± 2.1%. The findings of this study indicate that EEG signals can be used as an appropriate tool for measuring the cognitive load introduced by educational videos. This can be help instructional designers to develop more effective content.

## Introduction

Cognitive load is defined as the load being imposed on working memory while performing a cognitive task (Paas et al., 2004). There are three types of cognitive load: intrinsic, which is dependent on the nature of the task and cannot be modified by the designer; extraneous, which is related to the design of the task and can be altered by formatting the materials being presented; germane load which is associated with the amount of mental effort for building the schema in working memory (Sweller et al., 2019). Cognitive load assessment has a critical role in different areas such as education (Sweller, 2018) and human-computer interaction (HCI) designing (Zagermann et al., 2016). Multimedia plays an essential role in modern education. Keeping the amount of cognitive load at an optimum level is crucial in instructional design (Mutlu-Bayraktar et al., 2019). Mayer (2002), in his book Multimedia Learning, introduced twelve principles that help multimedia designers to minimize the amount of cognitive load on learners. Among these principles, five of them are devoted to extraneous processing, a type of cognitive processing in instructional multimedia learning, originating from the extra material in multimedia without any relevance to the instructional goal. The five principles for reducing extraneous processing are (1) *Coherence Principle*: extraneous words, images, and sounds should be excluded (e.g., attractive but non-related images); (2) *Signaling Principle*: essential materials should be highlighted with a cue (e.g., color highlight); (3) *Redundancy Principle*: in the presence of graphics and narration, the on-screen text should be excluded; (4) *Spatial Contiguity Principle*: corresponding words and images should be presented near to each other; (5) *Temporal Contiguity Principle*: corresponding words and images should be presented simultaneously, not successively. The effect of the introduced rules on cognitive load has been investigated based on behavioral, self-reported, and performance test data (Mayer and Mayer, 2005).

Cognitive load can be measured in five levels, within or between distinct tasks: overall, accumulated, average, peak, and instantaneous load (Antonenko et al., 2010). Instantaneous load reflects the amount of imposed cognitive load in each moment of a cognitive task (Antonenko et al., 2010). In general, there are two methods for cognitive load assessment: subjective [e.g., NASA-TLX questionnaire (Hart and Staveland, 1988)], and objective [e.g., electroencephalography (EEG) (Antonenko et al., 2010), eye-tracking (Pomplun and Sunkara, 2003; Barrios et al., 2004; Chen et al., 2011; Kruger and Doherty, 2016; Dalmaso et al., 2017; Latifzadeh et al., 2020), and fMRI (Tomasi et al., 2006)]. Subjective methods which are based on self-reporting have limitations for instantaneous or online assessment of cognitive load, and they are mainly being used for overall and average assessment of mental workload (Anmarkrud et al., 2019). In contrast, physiological measurements as objective methods have the advantage of measuring the cognitive load continuously and online during a cognitive task (Antonenko et al., 2010), such as video-based learning.

Electroencephalography as a neurophysiological measure with a high temporal resolution (approximately 1 ms) is a well-suited candidate for the assessment of cognitive load in educational environments because this method is objective, non-invasive, and less restricted in comparison to other neuroimaging methods (Antonenko et al., 2010). Nowadays, many portable EEG devices can be easily used in classrooms for cognitive load assessment (Xu and Zhong, 2018). Moreover, it has a high temporal resolution which is a good property for the assessment of instantaneous cognitive load. This ability may provide the opportunity to monitor the dynamics of cognitive load on working memory during a cognitive task such as multimedia learning. During the past decades, cognitive load has mainly been measured using subjective methods and behavioral data such as reaction times and error rates to perform specific tasks. According to the literature, EEG band power spectra (i.e., delta, theta, alpha, and beta) at different brain regions have been introduced to assess cognitive workload. Specially, theta and alpha have been linked to cognitive workload studies (Mazher et al., 2017; Puma et al., 2018; Castro-Meneses et al., 2020).

Several recent studies have empirically examined the relationship between cognitive demands and EEG activity at different frequency bands and brain regions. These studies have used EEG, alone or along with other subjective and objective measures, to assess participants’ cognitive workload in different environments, including performing the arithmetic task (Borys et al., 2017; Plechawska-Wójcik et al., 2019), engaging in a virtual reality space (Dan and Reiner, 2017; Tremmel et al., 2019; Baceviciute et al., 2020), and being in a multitasking situation (Puma et al., 2018). Moreover, most studies utilized statistical analysis to assess cognitive states/conditions based on subjective, behavioral, and physiological measure (Baceviciute et al., 2020; Castro-Meneses et al., 2020; Scharinger et al., 2020). However, recent studies have been focused on the usage of machine learning methods to improve the performance of cognitive load measurements (Plechawska-Wójcik et al., 2019; Appriou et al., 2020; Rojas et al., 2020).

Borys et al. (2017) applied several classification methods on different combinations of EEG and eye-tracking features to classify cognitive workload states on arithmetic task. They calculated power spectra of three frequency bands (theta, alpha, and beta) acquired from five scalp locations (Cz, F3, F4, P3, and P4) as EEG features. Their results showed that none of the EEG features were used in the best classification model. One limitation of this research was concentration on the specific brain regions with low effect in reducing workload. In a study carried out by Dan and Reiner (2017), they focused on EEG-based measures for cognitive load assessment related to event processing in 2D displays against 3D virtual reality environments. They calculated the ratio of the average power of the middle frontal theta (Fz) and the central parietal alpha (Pz) as cognitive load indicator. They found that the cognitive load of processing 3D information is lower than 2D. In a subsequent study, Tremmel et al. (2019) evaluated the feasibility of passive monitoring of cognitive workload*via*EEG while performing a classical n-back task in an interactive VR environment. They extracted EEG spectral powers of four frequency bands (theta, alpha, beta, and gamma) from eight electrode positions (Fz, F3, F4, C3, C4, P3, P4, and Pz). The Results revealed the positive correlation of alpha activity in the parietal area with workload levels. In another experimental paradigm, Puma et al. (2018) used theta and alpha band power to assess cognitive workload in a multitasking environment. In this task, the participants completed a task commonly used in airline pilot recruitment, with an increasing number of concurrent sub-tasks from one phase to the next phase of the task. They conducted their EEG analysis only on five electrodes centered in the frontal area (Fz, F3, F4, F7, and F8) for the theta rhythm and five electrodes centered in the parietal area (Pz, P3, P4, P7, and P8) for the alpha rhythm. Besides these EEG features, the researchers collected performance, subjective (NASA-TLX) and pupillometry measurements as overall cognitive workload indicators. According to the results, the power of both theta and alpha bands increased with task difficulty, indicating the direct effect of these bands in cognitive load. Although different indicators have been proposed in the literature, it is essential to explore the most optimal indices for assessing cognitive load in a specific research area such as multimedia learning environments.

In addition, there are a few studies on using EEG for cognitive load assessment in multimedia and video-based learning. Wang et al. (2013) used EEG frequency bands to classify two videos labeled confusing and non-confusing based on the participants’ self-reported feelings. They obtained an accuracy of 0.67 using a Gaussian Naïve Bayes classifier. In another study, Mazher et al. (2017) displayed identical video-based multimedia to their participants in three different sessions followed by a performance test. They assumed that by repeating the same content, cognitive load decreases. They also divided EEG signals into 10 s sections as the samples of their study. Using partial directed coherence (PDC) and support vector machine (SVM) classifiers, they inferred that the alpha band in the frontal and parietal lobes of the brain cortex could be a good indicator of cognitive load in multimedia learning. Lin and Kao (2018) showed that using Power Spectral Density (PSD) of all channels in EEG signal can discriminate different levels of mental effort in online educational videos. They examine three other models, including ANN, SVM, and decision tree. In a recent study, Castro-Meneses et al. (2020) assigned different levels of cognitive load based on the linguistic complexity of the presented content. They showed that theta oscillations are potentially an objective indicator of cognitive load.

In comparison to the previous related works, we follow an approach to reach the most informative brain regions and frequency bands associated with cognitive load. We assume that multimedia learning is a complex task in which different parts of the brain and may be different frequency bands are involved. Thus, it is hard to claim that only one or two regions of the brain in specific bands are important for measuring cognitive workload. Furthermore, we try to simulate the different conditions of instantaneous cognitive load in instructional videos by applying/violating the principles of multimedia which has rarely been attempted in the previous related works. We also investigate different time windows to find the optimal time frame for cognitive load assessment.

In this study, we aim to quantitatively measure the instantaneous cognitive load in multimedia learning using EEG signals. To this end, we design an experiment by applying/violating multimedia design principles to have two levels of cognitive load. Then, we build a classification model on the most informative spectral features. Using this model, we reach the goal of this manuscript, instantaneous cognitive load assessment. The rest of the manuscript is organized as follows. In the next section, we describe the materials of our study, including the educational videos, and the procedure of the experiment, and the methods that have been applied in our analyses. In section “Results,” we report the results of the current study, and finally, in section “Discussion,” a discussion on the results will be provided.

## Materials and methods

### Participants

**FIGURE 1:**
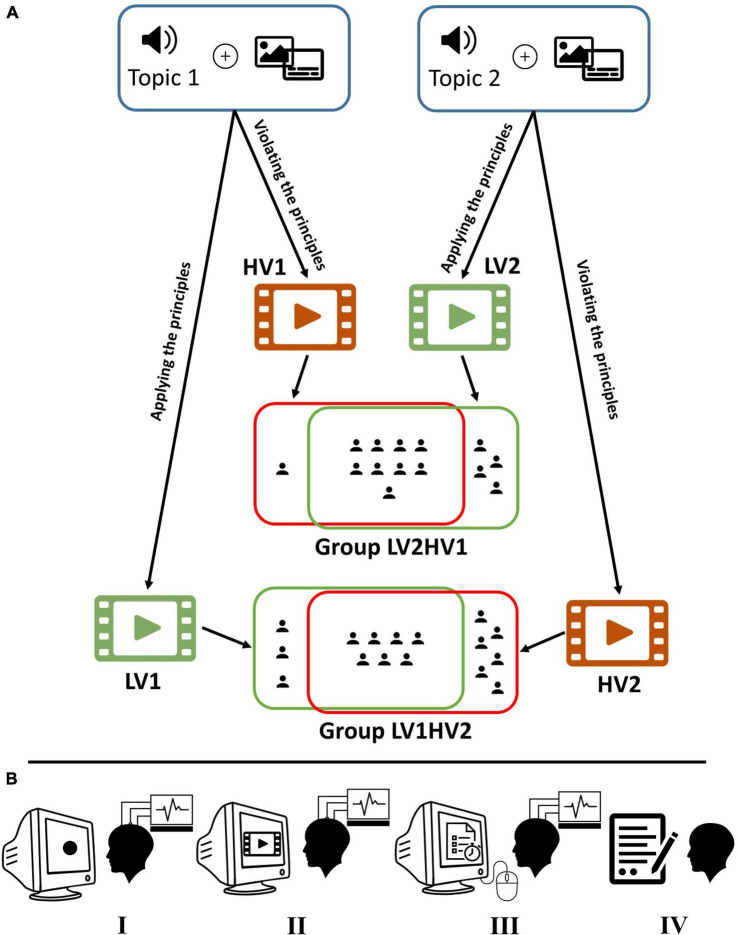
Experiment design. **(A)** Based on two audio narrations, four versions of videos (two for each narration) were created: LV1 (HV1) and LV2 (HV2) by applying (violating) the principles of multimedia design. Low load and high load video identified by green and red, respectively. Participants are randomly divided into two groups: LV1HV2 and LV2HV1. In our experiment, the LV1HV2 (LV2HV1) watched LV1 (LV2) and HV2 (HV1) videos in two separate sessions. As illustrated in the figure, in each group, some subjects participated only in one session. **(B)** The procedure of the experiment (left to right): first, looking at a black-filled circle for recording baseline data; second, watching the multimedia (no interaction); third, taking part in the recall test (via mouse interface); and finally completing the NASA-TLX questionnaire (paper-based version). In the first and second steps, electroencephalography (EEG) signals are collected.

Thirty-six university students between the ages of 18 and 25 participated in our experiment. Except for two, all other participants were male. The data acquired from six of them were discarded due to failure in recordings. The final set of our subjects includes thirty participants. We only excluded participants whose data were entirely corrupted. Thus, we tried to preserve as much data as possible for analysis. They are divided into two groups randomly to perform the task in two separate sessions. According to Figure 1A, 16 of them are in group 1 (LV1HV2) and 14 in group 2 (LV2HV1). Unfortunately, some participants participated only in one session and refused to continue the experiment due to their preferences. Thus, nine participants from group 1 and five participants from group 2 only watch one multimedia (see Supplementary material for detailed information about data management approach). The native language of all participants is Farsi (Persian), all of them are in the range of 23–32 in terms of listening skills of English which is evaluated by simulating the listening part of the International English Language Testing System (IELTS) exam. All participants were right-handed and had normal or corrected-to-normal eye vision. All participants signed informed written consent before attending the study. The experimental protocols were approved by the ethics committee of the Iran University of Medical Sciences.

### Educational multimedia

**FIGURE 2:**
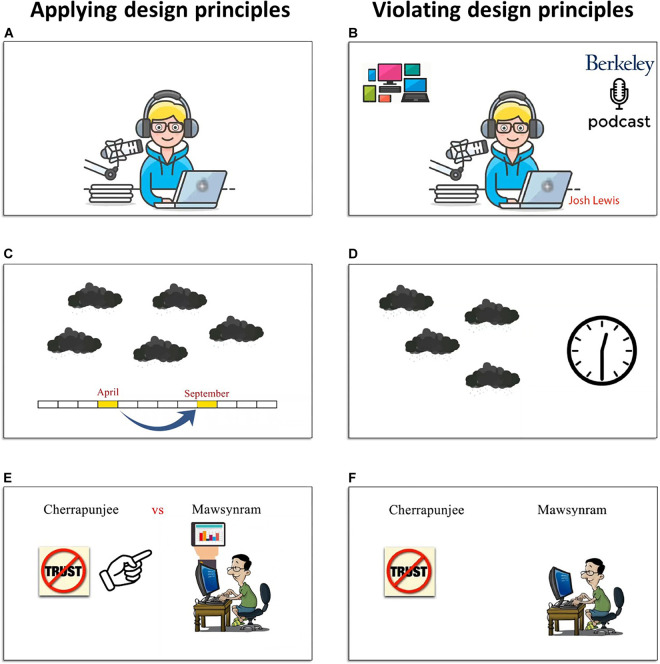
Educational multimedia. Examples of application (i.e., left panels: **A,C,E**)/violation (i.e., right panels: **B,D,F**) of multimedia design principles.

We created four multimedia. In two of them, we apply the multimedia design principles to impose a minimum amount of extraneous cognitive load on our participants. In contrast, the other two multimedia are created by violating these principles to impose a higher amount of cognitive load on the subjects in our study. We selected two chapters of Open Forum 3 (Duncan and Parker, 2007) which are listening comprehension tasks; lesson 6 and lesson 11 (for online access to the resources, see https://elt.oup.com/student/openforum/3?cc=ir&selLanguage=en hosted on Oxford University Press). Using the audio of each lesson, we created two versions of motion-graphic-animation (low-load and high-load) as two multimedia for that lesson (see Figure 2). The videos corresponding to lessons 6 and 11 have the length of 290 and 342 s, respectively. Two linguists in English language teaching devised the scenario for making the instructional videos and arranging the materials (texts and images). Then, all four videos have been created by a motion graphic specialist in Adobe After Effects CC 2017 v14.2.1.34 environment. We name the low-load versions of lesson 6 and lesson 11 as LV1 and LV2, respectively. Also, the high-load versions of lesson 6 and lesson 11 are named HV1 and HV2, respectively.

### Recall test and subjective questionnaire

We designed a multiple-options-question (MCQ) as a computer-based recall test with twelve identical questions for LV1 and HV1 and twelve identical questions for LV2 and HV2. The recall test has been designed by two linguists in the field of English language teaching. In addition to the recall test, we use the classic paper-based version of NASA-TLX (Hart and Staveland, 1988) as a subjective measure to compare the overall cognitive load between two conditions (i.e., low-load and high-load) in our study. NASA-TLX is a self-report index of cognitive load in the range of 0–100. Although the NASA-TLX is often used to measure general workload, a study (Mutlu-Bayraktar et al., 2019) that systematically reviews the cognitive load research literature in multimedia learning environments introduces NASA-TLX as a subjective indicator and performance outcomes as an indirect objective indicator for assessing cognitive load (the detailed information about NASA-TLX subscale values is provided as Supplementary material).

### Baseline

Before performing the main experiment, all subjects are requested to look at a black-filled circle (*r* = 5 mm) at the center of a gray screen for approximately 20 s. They are asked to keep relax and not think about anything special. We record EEG signals during this task and use the middle 10-s of the signals as our baseline in the analysis.

### Experiment design

After setting up the EEG cap on the participant’s head by a technician, the recording was started. The participant was alone in the semi-dark room, sitting 57 cm away from a 17-inch monitor with a refresh rate of 60 Hz. After a few seconds, when a timer in the center of the screen ends, the multimedia was played automatically. We asked participants to pay attention to the concepts presented in the video. There was no interaction between the person and the computer during the playing video. A few seconds after the multimedia is over; the recall test was started automatically. The participants could answer the questions in 420 s*via*a mouse interface. Participants had this option to leave any question unanswered. Moreover, there was the feasibility of moving between questions at any time, but only one question with all its options was displayed on the screen at a time. Also, the subject could terminate the recall test before the end of the timer. But by stopping the timer, the test phase was being finished automatically. The software platform for presenting the multimedia and recall test has been written in Java (for more details, see https://github.com/K-Hun/multimedia-learning-hci hosted on GitHub). After these steps, the EEG was stopped, and then the paper-based NASA-TLX was given to them. To make sure participants are familiar with the procedure and software environment of the experiment, we designed a trial phase before the experiment. In the trial phase, EEG signals are not recorded and also the multimedia is a 1-min video that is quite different in content and topic from the main multimedia of the experiment.

We assigned all thirty participants into two groups randomly, called LV1HV2 and LV2HV1 groups. Each subject participated in two distinct sessions of the experiment. The conditions in each group were counterbalanced across participants. Subjects in the LV1HV2 (LV2HV1) group performed the experiment in a session with LV1 (LV2) multimedia and in another session with HV2 (HV1) (some starting with the low load condition, and others with the high load one). Using this arrangement, each participant will not observe two multimedia with the same topic and audio and thus the concept of each multimedia is new to her/him. We summarized the experiment procedure in Figure 1.

### Electroencephalography recording and preprocessing

**FIGURE 3:**
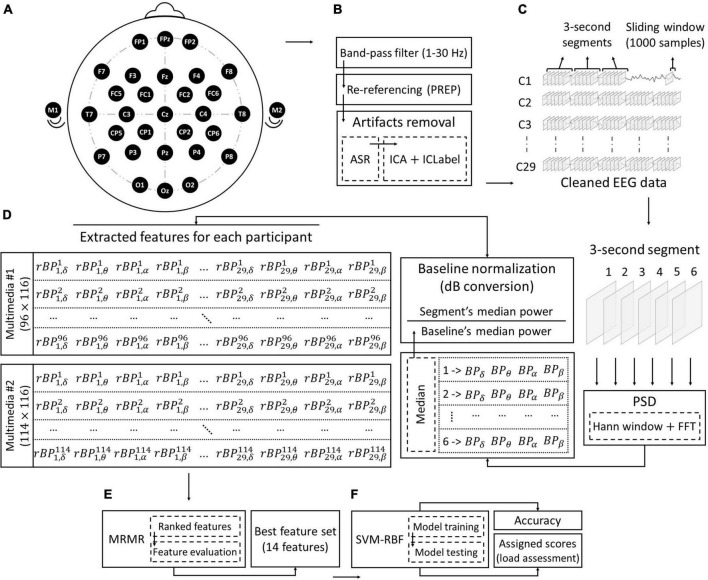
Electroencephalography (EEG) analysis workflow. **(A)** EEG acquisition: data collected from 29 channels for each participant during displaying the multimedia. **(B)** Pre-processing: includes band-pass filter, re-referencing, and artifacts removal processes. **(C)** Data segmentation: a sliding window (size = 1,000 *ms*; 50% overlappings) moves on the signal of each channel. Each of the six adjacent windows forms a 3s segment. **(D)** Feature extraction: by performing Hann window and by using the FFT method, PSD of all frequency sub-bands is calculated for each window. Next, the ratio of the median power of each 3s segment to the median power of the baseline is considered as the relative band power (*rBP*) of that segment. For each *rBP*, the superscript shows the segment number and the subscripts show the channel number and the band, respectively. Finally, the extracted features of each participant for each of the multimedia will be formed in 96 × 116 and 114 × 116 dimensions for multimedia 1 (i.e., LV1 and HV1) and multimedia 2 (i.e., LV2 and HV2), respectively. **(E)** Feature selection: the best set of features will be selected by evaluating the importance of the features which is ranked by the MRMR algorithm. **(F)** Classification: an SVM (kernel: RBF) is built to assign a score to each segment (assessment of instantaneous cognitive load).

To collect EEG data, we use a portable 32-channels eWave amplifier (Karimi-Rouzbahani et al., 2017a,b; Shooshtari et al., 2019) paired with eProbe v6.7.3.0 software. In this study, we recorded EEG data from 29 passive wet electrodes (FP1, FP2, FPz, F3, F4, F7, F8, Fz, FC1, FC2, FC5, FC6, C3, C4, Cz, T7, T8, CP1, CP2, CP5, CP6, P3, P4, P7, P8, Pz, O1, O2, and Oz) according to the 10–20 system of electrode placement, plus two bilateral mastoids (M1: left and M2: right) as the online reference for EEG signal potentials (see Figure 3A). The system has 24-bits data resolution with capturing 1K samples per second. Electrode impedances were kept below 5 KΩ in all recordings and electrode sites.

Analysis of EEG data and preprocessing are performed using the EEGLAB Toolbox v2020.0 and scripting in the MATLAB (R2019b) environment as shown in Figure 3B. As the first step, the basic FIR band-pass filter in the range of 1–30 Hz is applied to remove DC and high-frequency noise. Mastoid referencing makes EEG signals prone to external experimental artifacts. These artifacts come from the unstable connection of the EEG sensor to the mastoids, generating large spikes that are several orders of magnitude more prominent than the neural response produced by EEG. Therefore, in the next step, to reduce the effect of these artifacts, we apply the re-referencing part of the PREP pipeline algorithm (Bigdely-Shamlo et al., 2015) to estimate the true reference. Next, we utilize the Artifact Subspace Reconstruction (ASR) algorithm (Mullen et al., 2013) to correct corrupted parts of EEG data. ASR is being used to detect and remove high-amplitude components such as eye blinks, muscle movements, and sensor motion (Mullen et al., 2015). We perform ASR using *Clean_Rawdata* plug-in with default settings. A visual examination of the signals indicates that there are still some artifacts related to eye movements in the data. Thus, in the last step of preprocessing, independent component analysis (ICA) is applied using fastICA algorithm and the remaining artifacts (i.e., eye movements) are removed from the data using IC Label with threshold of 90% (Pion-Tonachini et al., 2019).

### Segment length analysis

**FIGURE 4:**
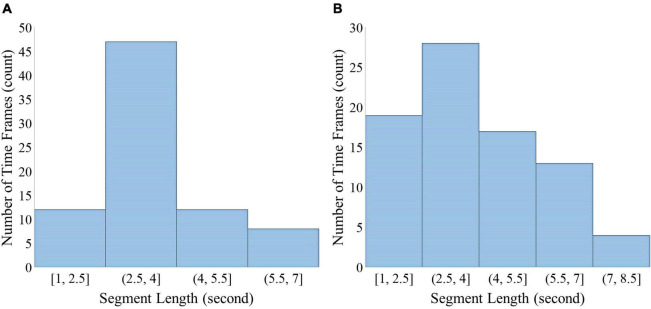
Segment length analysis in **(A)** multimedia 1 and **(B)** multimedia 2. Histograms show the frequency of number of meaningful timeframes regarding segment length for multimedia 1 **(A)** and multimedia 2 **(B)**.

One challenge in the assessment of instantaneous cognitive load is selecting the most appropriate segment length. This issue has not been clearly answered in the previous related studies, so different time interval has been adopted as segment length. Here, we are faced with a content-oriented task (i.e., multimedia learning). To this end, we are seeking to achieve the smallest meaningful and informative interval in the multimedia learning task by analyzing the optimal time window selection. Hence, we consider the average time spent to convey a meaningful phrase to learners as a metric to determine the segment length. For this purpose, we use the silent moments in the audio narrations of the multimedia as an appropriate situation for learners to understand the contents presented before these moments. We use WavePad Sound Editor v12.4 to find silence points with minimum duration of 300 ms and below 25 dB level. The segments with audio narration that conveys some words without silent interruptions in two multimedia are shown in Figure 4. The figure illustrates the number of time frames with audio narration for each segment length. As shown in this figure, it is desirable to choose a segment length in the range of 2.5–4 s. Thus, in the following, we assess the instantaneous cognitive load for segments with a length of 3 s.

### Feature extraction

We adopt a time-frequency-based analysis approach for feature extraction. For each participant’s EEG data, the PSD in each channel is estimated by calculating the squared magnitude of the fast-Fourier transform (FFT) (Semmlow, 2011) from 50% overlapping windows, which is tapered by the Hanning window to reduce the spectral leakage. A window size contains 1,000 sample points (1 s) and an overlap of 500 sample points (500 ms) (see Figure 3C). Next, relative band power (*rBP*) of 3 s segments are extracted in each frequency band: delta (δ:2–3 Hz), theta (θ:4–7 Hz), alpha (α:8–12 Hz) and beta (β:13–30 Hz). In order to extract these frequency bands, for each segment, we performed the decibel (dB) conversion (Cohen, 2014). The dB conversion is a baseline normalization method that quantifies the ratio of the median PSD in each band and the median PSD of the baseline on a logarithmic scale. In this way, we overcame the positively skewed distribution of EEG power data. By applying this method, power values are often normally distributed and thus parametric statistical analysis is an appropriate approach for feature extraction (Cohen, 2014).

To calculate the *rBP*, we use Eq. (1) where $r\,B\,P_{c\,h,b}^{i}$ is the median power of *i*-th segment *seg* in the channel *ch* (*ch* ∈ {1, 2,…, 29}) and the band *b*(*b* ∈ {δ,θ,α, β}) relative to the median power of the baseline *base* in same channel and band. Moreover, *seg*_*i*_ indicates EEG data of the *i*-th segment.


(1) $$r\,B\,P_{c\,h,b}^{i}=10\,l\,o\,g\,10\,\left(\frac{m\,e\,d\,i\,a\,n\,P\,S\,D_{c\,h,b}^{s\,e\,g_{i}}}{m\,e\,d\,i\,a\,n\,P\,S\,D_{c\,h,b}^{b\,a\,s\,e}}\right)\;$$

By concatenating the extracted features for the *i*-th segment, a feature vector (*FV*_*i*_) is constructed for that segment. This feature vector consists of 116 elements (4 *rBP*s in 29 channels), as follows:


(2) $$FV_{i}=[rBP_{1,\delta }^{i},rBP_{1,\theta }^{i},rBP_{1,\alpha }^{i},rBP_{1,\beta }^{i},\ldots ,rBP_{29,\delta }^{i},rBP_{29,\theta }^{i},rBP_{29,\alpha }^{i},rBP_{29,\beta }^{i}]{}_{1\times 116}$$

Extracted features of each participant in all segments are illustrated in Figure 3D.

### Feature selection

In the next step, we select the best discriminative feature set with the highest prediction accuracy. Also, it is essential to determine the regions of the brain and frequency bands that are highly informative for predicting cognitive load. To address this goal, we use the minimum redundancy-maximum relevance (MRMR) algorithm (Peng et al., 2005), which is a mutual information-based feature selection method. The algorithm follows an incremental search method iteratively. At each iteration, the candidate feature will be examined whether it has: (1) maximum relevance with respect to the class label, and (2) minimum redundancy with respect to the features selected at previous iterations. To evaluate the importance of features, a score is calculated for each feature according to these two criteria. Next, the MRMR algorithm will rank the features based on the scores in descending order. This process returns the ranking of 116 features which indicates the importance of each frequency band and channel. However, the limitation of this process is that the best feature set is not determined, and the optimal feature set must be selected by evaluating the ranked list with respect to the classification performance. To this end, we evaluated the ranked features by applying Linear Discriminant Analysis (LDA) (McLachlan, 2004) to samples in the following manner, to achieve the best set that improves the performance of classification. At first, the samples of all segments are split into 10 folds such that one fold is considered as the test set and the remaining folds are used to train the LDA model. Then, by increasing the number of features for every sample from 1 to 116 according to the ranking obtained by the MRMR algorithm, the LDA model is trained using selected features and prediction accuracy is computed on the test set. This process is repeated 10 times by considering each fold as a test set. Finally, by averaging over prediction accuracy of different folds, the final accuracy is computed for a subset of features (from 1 to 116) (see Figure 3E).

### Classification of cognitive load

In this phase, in order to assess the instantaneous cognitive load, we follow an approach that classifies segments into two conditions (i.e., low-load and high-load). Our goal is to assign a score of cognitive load to each 3 s segment based on the distances between the samples and decision boundary (see Figure 3F).

To perform classification and assign scores to segments, we use the SVM algorithm. The algorithm has been widely used for non-linear binary classification problems in machine learning. It has achieved desirable results in cognitive and mental task applications (Amin et al., 2017). SVM transforms input data into higher dimensional space by applying the kernel trick, after which it finds the hyperplane with the best generalization capabilities by maximizing the margins (Wang et al., 2009). SVM with the kernel is extremely sensitive to hyperparameters, so it must be tuned to achieve a good level of performance. Hence, we apply the radial basis function (RBF) kernel, which only needs to optimize two hyperparameters (i.e., C as the penalty parameter and γ as the kernel width parameter) (Hsu et al., 2003). We examine various pairs of (C, γ) values using the Bayesian optimization algorithm, and the one set with the lowest cross-validation loss is selected. In the next step, in order to measure the performance of the optimized classifier and extract classification scores, we randomly select 70% of samples as a training set, and the rest of the samples are considered as a test set. Then, the classification scores are computed as mental workload scores. Indeed, these scores indicate the signed distance between a sample and the decision boundary. The score (*s_i*) for *i*-th segment is computed as follows:


(3) $$s_{i}=\sum_{j=1}^{n}p_{j}\,y_{j}\,G\,\left(s\,v_{j},s\,e\,g_{i}\right)+q$$

where *G*(*sv*_*j*_,*seg*_*i*_) is a non-linear transformation with radial basis function (RBF) which is defined in Eq. (4).


(4) $$G\,\left(s\,v_{j},s\,e\,g_{i}\right)=\exp\left(-{||s\,v_{j}-s\,e\,g_{i}||}^{2}\right)$$

where *n* is the number of support vectors, *sv*_*j*_ is *j*-th support vector, *y*_*j*_ ∈ {−1,1} (i.e., low-load: –1 and high-load: +1) is the label of *j*-th support vector, *p_j* is the estimated SVM parameter for *j*-th support vector and *q* is the bias term. For more details on the estimation of (*p*_1_,…,*p*_*j*_,*q*) see Cristianini and Shawe-Taylor (2000).

Three values of the score (*s*) would be possible based on the position of each sample: (1) zero value (*s* = 0) when the sample is located on the decision boundary (hyperplane); (2) positive value (*s* > 0) when the sample has been correctly classified; (3) negative value (*s* < 0) otherwise. Once the scores are determined, we will normalize them to the range of 0–1 using the min-max normalization method as follows:


(5) $$S\,C_{i}=\frac{s_{i}-\text{min}\,\left(S\right)}{\max\left(S\right)-\min\left(S\right)}$$

where *s_i* and *S* are the scores of the *i*-th segment and the set of all segments’ scores obtained after SVM classification, respectively.

## Results

In this section, first, we validate the experimental conditions. Second, we examine the appropriate time interval for assessing the cognitive load imposed by the educational videos. Third, we evaluate the selected features and identify the most important frequency bands and brain regions for distinguishing two mental workload conditions. Finally, we present the results of the scoring model for instantaneous cognitive load assessment and investigation of its generalizability.

### Validation of experimental conditions

**FIGURE 5:**
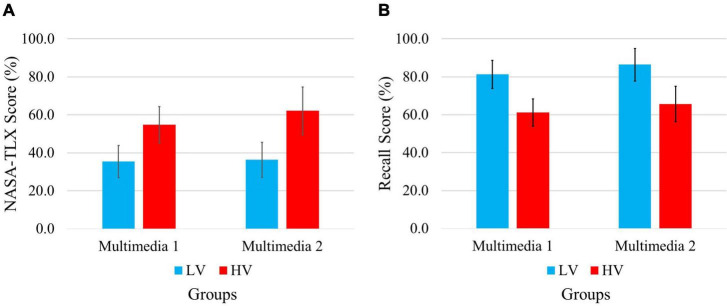
Comparison of **(A)** NASA-TLX scores and **(B)** recall test scores in two experimental conditions. In each graph the scores [NASA-TLX scores in panel **(A)** and Recall scores in panel **(B)**] are compared between two conditions (LV and HV) for each multimedia (Multimedia 1 and Multimedia 2). The scores have been scaled in the range [0, 100].

To validate two experimental conditions (i.e., low-load and high-load), we performed statistical analysis on NASA-TLX scores and recall test. The assumption is that applying/violating multimedia design principles imposes different levels of cognitive load on learners. As a result, a two-sided independent samples t-test was used to investigate statistical differences for the two experimental conditions. The average and standard deviation of NASA-TLX scores and recall test scores in each group are presented in Figure 5. This analysis on NASA-TLX scores indicates a significant difference between cognitive load imposed by the different instructional design in multimedia, *t*(18) = −4.87,*p* < 0.0002 and *t*(24) = −6.07,*p* < 0.0001 for multimedia 1 (i.e., LV1 and HV1) and multimedia 2 (i.e., LV2 and HV2), respectively. Also, the same analysis on recall test shows that *t*(18) = 6.41,*p* < 0.0001 and *t*(24) = 6.22,*p* < 0.0001 for multimedia 1 and multimedia 2, respectively. Thus, two groups in both multimedia have significantly different performances. These results validate the assumption that the different mental demands are elicited due to the experimental conditions.

### Evaluation of selected features and activated cerebral regions

The goal of feature selection is to extract the optimal feature set by reducing redundancy while keeping the information of gathered data. After performing the method described in section “Feature selection,” we select the top 14 features of the MRMR algorithm as the best subset. This feature set can achieve the highest classification accuracy of 78.34 ± 1.3% using the LDA method for two load conditions. The best feature set is ordered in Eq. (6), where each element represents the selected channel with the band in the subscription.


(6) $$BestFeatures=\{O1_{\alpha },C3_{\alpha },P3_{\theta },P7_{\theta },CP1_{\delta },P7_{\beta },O2_{\delta },FC5_{\alpha },CP1_{\beta },FPz_{\alpha },FC6_{\alpha },C4_{\theta },F7_{\alpha },F7_{\delta }\}$$

**FIGURE 6:**
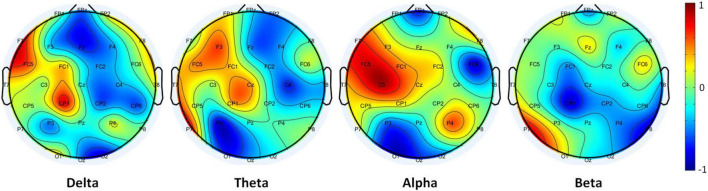
Differences between average relative band powers of electroencephalography (EEG) features (bands and locations) in two conditions.


For evaluating the selected features, we investigated the overall brain topographic difference between two experimental conditions in each frequency band. For this purpose, first, we calculated the average *rBP* [see Eq. (1)] of all 3 s segments of each condition (i.e., low-load and high-load) in each band and then subtracted the average of low-load average from the average of high-load. Figure 6 illustrates the difference between the *rBP* averages of two conditions in each band. The powers in each band are scaled to the range of −1 to +1. According to this figure, active cortical areas are different in each band, and we can determine active cerebral regions for each band as below where the superscription (i.e., L: low-load and H: high-load) indicates the corresponding condition.

(7) $$\delta_{A\,C\,T\,I\,V\,E}=\{F7^{H},CP1^{H},FC5^{H},FC2^{L},P3^{L},FPz^{L},CP2^{L},Oz^{L},CP6^{L},Fz^{L},O2^{L}\}$$

(8) $$\theta_{A\,C\,T\,I\,V\,E}=\{P7^{H},F3^{H},FC5^{H},T7^{H},T8^{L},Oz^{L},Fz^{L},FP2^{L},O1^{L},C4^{L},P3^{L}\}$$

(9) $$\alpha_{A\,C\,T\,I\,V\,E}=\{C3^{H},FC5^{H},F7^{H},P4^{H},FC1^{H},P3^{L},O2^{L},Oz^{L},FC6^{L},FPz^{L},O1^{L}\}$$

(10) $$\beta_{A\,C\,T\,I\,V\,E}=\left\{P\,7^{H},F\,C\,1^{L},P\,4^{L},T\,8^{L},F\,P\,z^{L},C\,P\,1^{L},P\,8^{L}\right\}$$

The results show that the selected features are consistent with the active cerebral regions in different locations and bands. It is inferred from the comparison of the best feature set [as mentioned in Eq. (6)] and the active cerebral regions [as stated above in Eqs (7–10)]. So that, all the selected features were selected from the active cortical areas. This indicates that the feature selection method effectively selects a combination of informative and relevant features to cognitive load with respect to the brain activity map.

**FIGURE 7:**
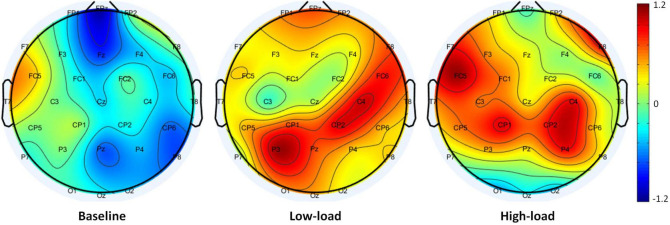
Brain topographies of alpha power distribution in two conditions compared to baseline. From left to right, the topographies represent the average relative alpha power for the eye-opened baseline, low-load, and high-load conditions.

In order to identify which frequency band can distinguish two cognitive load conditions more effectively, we perform the classification task in each frequency band separately by selecting the feature subset associated with that band. Again, we apply 10-fold cross-validation using the LDA method on data. As presented in Table 1, the alpha is the best frequency band for predicting mental workload. The predictive power of the alpha feature set is 73.85 ± 2.73%. Figure 7 illustrates brain topographies of relative alpha power distribution in two conditions compared to the baseline. According to this figure, the diagonal activity of alpha power in each condition attracts attention. In low-load condition, most alpha activation is concentrated in the left lateral posterior to the right lateral anterior cortices. Conversely, in high-load condition, this pattern is localized in the right lateral posterior to the left lateral anterior cortical areas. By comparing Eqs (6) and (9), it is found that alpha power suppression in prefrontal midline (*FPz*), right lateral frontal (*FC6*), and left lateral occipital (*O1*) cortices have a more significant impact on increasing cognitive load. Also, activation of alpha power in the left lateral frontal (*FC*5,*F*7) and left central (*C*3) cortical areas synchronize by increasing cognitive load.

**TABLE 1:** Classification accuracy and standard deviation (std) of electroencephalography (**EEG**) **band powers using** linear discriminant analysis (**LDA**).

		Frequency bands		
	**Delta (δ)**	**Theta (θ)**	**Alpha (α)**	**Beta (β)**

**Accuracy**	72.97	68.33	73.85	68.22
**Std (±)**	2.29	2.23	2.73	2.23

Results are presented in percentage.

### Instantaneous cognitive load scoring model results

After evaluating the best feature set, we evaluate the performance of the classification method presented in section “Classification of cognitive load” for assigning scores to segments. Thus, we compute the average and standard deviation of classifier accuracy to assess the SVM model performance. The performance of the model is achieved 84.5 ± 2.1%. As described previously, assigned scores are converted into normalizing scores (*SC*s) using Eq. (5).

**FIGURE 8:**
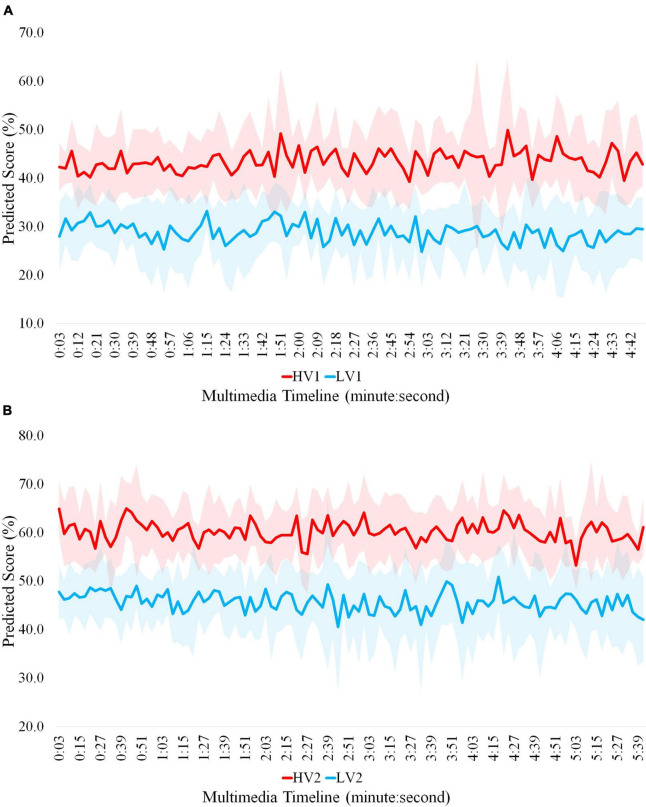
Predicted cognitive load scores in **(A)** multimedia 1 and **(B)** multimedia 2 over time.

Then, the cognitive load imposed at each moment of each multimedia is calculated by averaging over the normalized scores obtained by the SVM in the corresponding segments at that moment. Figure 8 displays predicted workload scores in two multimedia over time. As depicted in this figure, the average of predicted scores corresponding to two load conditions is significantly different across multimedia timeline. These scores for LV1, HV1, LV2, and HV2 are 29, 43, 46, and 60, respectively.

## Discussion

In this study, based on the most informative feature set, we construct an SVM model for assessing instantaneous cognitive load. To impose low or high levels of cognitive load on the participants, we designed an experiment with two versions of multimedia by applying or violating the principles of multimedia design. The conditions of our experiment are evaluated by a recall test and a NASA-TLX as a subjective measurement of cognitive load. As a result, applying the principles leads to lower NASA-TLX scores and improvement of performance tests, indicating that this experimental condition will induce a lower cognitive load in comparison to the condition of violating design principles.

In order to extract the informative and relevant EEG features as an objective measurement of cognitive load, first, we calculated the PSD of the common frequency bands. Then, we extracted the optimal feature set by using the MRMR algorithm, which is a ranking method based on mutual information. The main advantage of this feature selection method is the effective reduction of redundant features while preserving relevant features. In addition, compared to other dimensionality reduction techniques such as PCA, the readability and interpretability of the features are held, and no changes are made to the data.

The selected feature set includes less than 12% of the total features. These 14-top features confirmed the different conditions of the cognitive load imposed on the subjects very good. The selected features show a remarkable combination of activated frequency bands in different brain regions associated with executive functions of brain which are referred to as supervisory cognitive processes (e.g., attention, cognitive inhibition, or learning) because they involve higher level organization and execution of complex thoughts and behavior (Alvarez and Emory, 2006; Whelan, 2007). Especially, in multimedia learning, verbal information (e.g., words or sentences) and visual part (e.g., illustrations, photographs, or diagrams) are merged (Gyselinck et al., 2008). These audio/visual signals may arise conflicting effects and overloads on the overall brain, and thus it is expected to have a simultaneous activation of different areas of the cerebral cortex. Given the specified locations/frequencies, it can be possible to find cognitive load differences at these locations/frequencies using simple statistical analysis.

Most of the selected features are from the frontal region (*FPz*_α_, *FC*5_α_, *FC*6_α_, *F*7_α_, and *F*7_δ_). Except for one of them (*F*7_δ_), the other mentioned features belong to the alpha band. In addition, two features have been selected from the centro-parietal (*C*3_α_) and the occipital (*O*1_α_) regions. This result is in line with previous studies that link cognitive processes to the frontal and parieto-occipital regions (e.g., Puma et al., 2018 for review), and alpha band activity (e.g., Foxe and Snyder, 2011 for review). According to the literature, activation of alpha indicate two opposite behaviors related to cognitive processing: active processing associated with memory maintenance and inhibition of irrelevant information (Jensen and Mazaheri, 2010). In fact, the increase in cognitive workload may be due to either of these two reasons or both of them. In this study, we observed that the power of alpha band in the low-load condition (i.e., applying design principles) is higher than the high-load condition (i.e., violating design principles), prominently in the prefrontal and the occipital regions. The increases of alpha spectral power seems to reflect the top-down control of the parieto-frontal attention network. As reported in recent studies, this mechanism inhibits irrelevant information flow from the visual perception system and internal cognitive processing (Pi et al., 2021). In this way, the information is transferred from task-irrelevant regions to task-relevant ones (Jensen and Mazaheri, 2010). Therefore, the decrease in alpha power near Broca’s area, which plays a significant role in language comprehension (Novick et al., 2005), suggests the effective engagement of cognitive resources related to the task.

After feature analysis, we propose a scoring model to measure instantaneous cognitive load in 3s segments of multimedia. The model can predict the mental workload scores in multimedia across time at appropriate accuracy. In other words, applying (violating) principles at each moment has caused that the predicted cognitive load score for LV1 (HV1) and LV2 (HV2) is lower (higher) than HV1 (LV1) and HV2 (LV2) at that moment. This allows us to monitor and manage learners’ cognitive status while watching multimedia at each moment. In this way, we can evaluate the quality of presented instructional materials and design principles in multimedia across time. Also, it can be possible to measure the effect size and impact of applying each principle. Therefore, by detecting the segments of multimedia that impose a great cognitive load on learners, we can provide the optimal load and improve learning performance by applying appropriate instructional materials and effective design principles. Moreover, a comparison of several multimedia that convey the same content can be feasible. This ability facilitates the production or selection of appropriate educational multimedia based on cognitive neurophysiological indicators.

Several limitations in the current research should be noticed. The first limitation of this study is the use of gel-based EEG equipment to collect data. The sensitivity of this device to get good contact of electrodes to scalp sites makes data prone to noise, resulting into extra time for preprocessing and increase in data loss rate. Moreover, for future studies, it might be useful to evaluate some cognitive-related abilities of subjects such as short-term memory capacity, visual attention, auditory and visual processing, etc. These abilities can be evaluated by common psychometric tests. In addition, it is a good idea to consider the cognitive and learning styles of participants in future studies. Another limitation to be mentioned here is the restriction of the analytical method. We assessed cognitive load by analyzing features extracted from the electrodes individually. Therefore, the interconnected functionality of the brain during a cognitive task is not considered. It is essential to consider the brain connectivity analysis approach in future researches to investigate information flows that are important in cognitive processes.

## Conclusion

In this study, we investigated the possibility of instantaneous assessment of cognitive load in educational multimedia using EEG data as an objective measure. Our experimental conditions, which impose two distinct levels of cognitive load by applying/violating multimedia design principles to learners, were validated by using the result of the NASA-TLX and recall test. We extracted the relative band powers for common frequency bands in each cerebral area. The most informative and relevant feature set for measuring cognitive load was selected using the MRMR method. We constructed an SVM classification model to predict cognitive load scores at 3s moments. The proposed model was validated for generalization from one multimedia to another. This capability can significantly help educational multimedia designers to construct multimedia by imposing an optimal amount of cognitive load on learners. In short, our main contributions in this study can be considered as (1) investigation of active cortical areas and major frequency bands associated with cognitive load in learning task, (2) instantaneous assessment of cognitive load in educational multimedia using objective indicators, and (3) generalizability of the workload scoring model from one multimedia to another.

## Data availability statement

The raw data supporting the conclusions of this article will be made available by the authors, without undue reservation.

## Ethics statement

The studies involving human participants were reviewed and approved by Ethics Committee of the Iran University of Medical Sciences. The patients/participants provided their written informed consent to participate in this study.

## Author contributions

RS and KL performed the experiment. KL implemented the HCI software platform. RS analyzed the data and wrote the manuscript under the supervision of SA and in consultation with AB. SA and AB determined the methodology, including signal processing methods and machine learning approaches. RE conceptualized and provided domain knowledge in this study and conducted the research direction. All authors contributed to the manuscript and approved the submitted version.
