# Supplementary material for: Assessment of instantaneous cognitive load imposed by educational multimedia using electroencephalography signals
Source: Front Neurosci. 2022 Aug 1;16:744737. doi: 10.3389/fnins.2022.744737 (PMC9377376; doi:10.3389/fnins.2022.744737)
Supplement: Supplementary file 1 [file Data_Sheet_1.pdf]

Supplementary Material

1 Data Management Approach

We prefer to use all correct data in data analysis. Therefore, we provided the results of all correct data in the main manuscript. However, it is possible to follow two different approaches for data analysis and compare their results with the results of all data to validate the soundness of the analysis. First, we applied the classification approach (as explained in the manuscript) on each multimedia, separately and computed the total model performance again. This strategy is called *"fully independent"* data analysis. Next, we removed participants who only experienced one condition of multimedia and computed the model performance again as *"fully dependent"* data analysis. We provide the performances of the proposed model in three conditions in Table S1. According to the results, the accuracy of the method in the two new approaches is close to the current approach.

Table S1: Comparison of the model performance in three approaches.

	**Current analysis**	**Fully independent analysis**	**Fully dependent analysis**
**Accuracy (%) $\pm$ Std**	84.5 $\pm$ 2.1	Multimedia 1: 87.45 $\pm$ 1.6<br>Multimedia 2: 86.24 $\pm$ 1.7<br>Average: 86.85	82.55 $\pm$ 3.6

2 NASA-TLX Subscale Values

In order to collect workload assessments with the NASA-TLX, the multi-dimensional rating procedure is used to calculate an overall workload score. This procedure provides the score based on a weighted average of ratings on six subscales (factors): 1) Mental demand, 2) Physical demand, 3) Temporal demand, 4) Effort, 5) Performance, and 6) Frustration. Each participant goes through two stages of evaluation: 1) rating, and 2) weighting each scale. The rating of each scale reflects the magnitude of that factor in the task, with the range from 0 to 100 in increments of 5. The weighting evaluates the contribution of each factor to the workload of the task. There are 15 possible pair-wise comparisons of the six subscales. The number of times that each factor is selected is counted as the weight of that factor. The weight can range from 0 to 5. In this way, the overall workload score for each participant is calculated by multiplying each rating by its weight given to that factor by that participant. Then, the sum of the weighted ratings is divided to 15. Detailed subscale values in each condition are presented below (from Table S2 to Table S5).

Table S2: NASA-TLX subscale values of Multimedia #1 LV (Low-load Video)

Subject	Mental	Physical	Temporal	Performance	Effort	Frustration	Score
**1**	70 (5)	10 (2)	40 (1)	35 (4)	50 (3)	10 (0)	46.67
**2**	60 (1)	65 (4)	75 (0)	25 (2)	55 (3)	30 (5)	45.67
**3**	45 (4)	15 (0)	30 (4)	35(4)	40 (1)	10 (2)	33.33
**4**	65 (4)	30 (2)	50 (1)	20 (5)	60 (3)	25 (0)	43.33
**5**	40 (2)	30 (5)	30 (3)	20 (0)	85 (1)	20 (4)	32.33
**6**	35 (2)	20 (5)	25 (0)	25 (1)	40 (4)	40 (3)	31.67
**7**	40 (4)	60 (2)	25 (1)	15 (3)	30 (2)	40 (3)	35.33
**8**	50 (5)	15 (0)	10 (2)	20 (4)	55 (2)	15 (2)	32.67
**9**	55 (3)	30 (1)	50 (2)	5 (5)	40 (4)	5 (0)	32.00
**10**	25 (3)	15 (1)	5 (2)	20 (5)	25 (4)	5 (0)	20.00

Weight of each factor is presented in parentheses.

Table S3: NASA-TLX subscale values of Multimedia #1 HV (High-load Video)

Subject	Mental	Physical	Temporal	Performance	Effort	Frustration	Score
**1**	40 (2)	60 (5)	50 (0)	70 (4)	75 (1)	25 (3)	54.00
**2**	50 (1)	25 (3)	55 (3)	20 (3)	75 (4)	20 (1)	44.67
**3**	85 (4)	15 (2)	65 (3)	75 (2)	55 (3)	70 (1)	63.33
**4**	85 (3)	60 (0)	80 (1)	75 (5)	75 (4)	15 (2)	69.33
**5**	15 (1)	75 (5)	40 (4)	15 (3)	25 (1)	25 (1)	43.00
**6**	30 (2)	40 (4)	15 (1)	80 (5)	45 (3)	45 (0)	51.33
**7**	65 (3)	60 (2)	40 (0)	80 (4)	65 (3)	70 (3)	69.33
**8**	25 (2)	25 (3)	25 (0)	80 (5)	65 (4)	20 (1)	53.67
**9**	70 (4)	10 (0)	65 (1)	35 (4)	40 (3)	55 (3)	51.33
**10**	25 (1)	60 (2)	45 (3)	35 (5)	60 (4)	35 (0)	46.33

Table S4: NASA-TLX subscale values of Multimedia #2 LV (Low-load Video)

Subject	Mental	Physical	Temporal	Performance	Effort	Frustration	Score
**1**	45 (4)	15 (2)	50 (0)	25 (4)	45 (2)	25 (3)	31.67
**2**	55 (3)	25 (1)	50 (0)	50 (5)	60 (3)	35 (3)	48.33
**3**	75 (5)	50 (2)	50 (0)	20 (4)	30 (3)	15 (1)	44.00
**4**	35 (0)	40 (4)	20 (3)	30 (1)	25 (2)	10 (5)	23.33
**5**	75 (2)	10 (3)	35 (4)	50 (1)	40 (3)	60 (2)	40.67
**6**	45 (4)	0 (0)	45 (1)	65 (3)	55 (3)	20 (4)	44.33
**7**	95 (3)	55 (0)	30 (4)	5 (4)	50 (3)	50 (1)	41.67
**8**	10 (2)	35 (5)	30 (4)	25 (1)	20 (1)	20 (2)	26.67

**9**	25 (3)	25 (5)	20 (0)	30 (4)	15 (2)	15 (1)	24.33
**10**	45 (3)	25 (2)	35 (1)	30 (5)	35 (1)	30 (3)	33.00
**11**	85 (1)	60 (1)	40 (4)	5 (4)	60 (3)	60 (2)	41.67
**12**	65 (4)	10 (0)	10 (1)	40 (5)	45 (3)	10 (2)	41.67
**13**	35 (2)	5 (4)	30 (2)	70 (5)	25 (2)	85 (0)	36.67

Table S5: NASA-TLX subscale values of Multimedia #2 HV (High-load Video)

Subject	Mental	Physical	Temporal	Performance	Effort	Frustration	Score
**1**	85 (5)	25 (1)	50 (2)	45 (4)	85 (3)	20 (0)	65.67
**2**	60 (2)	85 (5)	70 (0)	30 (3)	70 (4)	30 (1)	63.00
**3**	90 (5)	15 (0)	90 (1)	65 (4)	65 (2)	40 (3)	70.00
**4**	65 (3)	30 (5)	45 (1)	20 (1)	65 (1)	65 (4)	49.00
**5**	90 (3)	65 (0)	65 (1)	90 (5)	85 (4)	20 (2)	77.67
**6**	60 (2)	90 (5)	70 (0)	40 (3)	75 (4)	50 (1)	69.33
**7**	65 (4)	50 (4)	15 (2)	15 (1)	80 (4)	5 (0)	55.00
**8**	60 (2)	90 (5)	25 (0)	15 (3)	70 (1)	80 (4)	67.00
**9**	70 (3)	55 (3)	40 (1)	45 (3)	70 (2)	70 (3)	60.00
**10**	50 (5)	20 (0)	35 (1)	30 (4)	45 (3)	35 (2)	40.67
**11**	40 (3)	25 (5)	25 (1)	85 (4)	35 (2)	15 (0)	45.33
**12**	75 (2)	50 (4)	60 (3)	20 (1)	65 (2)	70 (3)	59.33
**13**	100 (4)	95 (2)	95 (3)	50 (3)	85 (3)	75 (0)	85.33

To investigate the effect of two factors of *“physical demand” and “temporal demand”* on the final scores of the NASA-TLX questionnaire, we calculated the scores without considering these two factors. We expected that the scores obtained would not be significantly different from the scores obtained from all factors. Because we believe that participants have been endured less physical and temporal demands than other factors (i.e., mental demand, performance, effort and frustration) to perform the task. Figure S1 shows a comparison between the scores corresponding to each of the multimedia in two conditions. Statistical analysis on the scores calculated in the two series indicated that there is no significant difference between the scores calculated from the *"All factors"* condition and the *"Without two factors"* condition, t (90) = -0.28 and p > 0.7. Also, two series are highly correlated, r = 0.89.

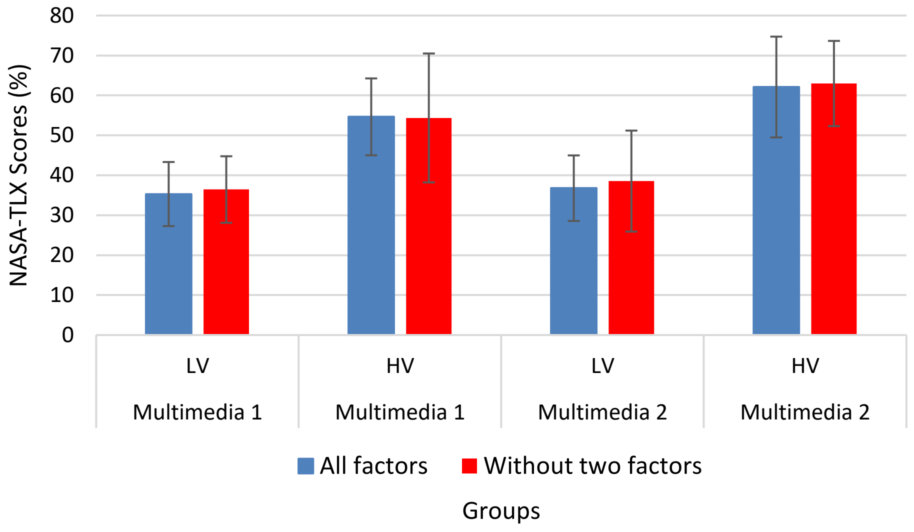

Figure S1. NASA-TLX scores in two conditions: (1) All factors, and (2) Without two factors (i.e. without “physical demand” and “temporal demand”). Each bar shows the mean and the standard deviation of the NASA-TLX scores in each group in two conditions.
